# How positive childhood experiences foster college students’ adjustment: the role of sibling number and self-esteem

**DOI:** 10.3389/fpsyg.2024.1305609

**Published:** 2024-02-14

**Authors:** Juan Li, Xiumei Zhang, Siqi Chang, Can Zhang, Wenzhuang Wang

**Affiliations:** School of Health Care Security, Shandong First Medical University, Jinan, China

**Keywords:** positive childhood experiences, China college students’ adjustment, siblings, self-esteem, mediating role, antagonistic moderating role

## Abstract

**Introduction:**

In recent years, growing concern has emerged regarding the mental health and well-being of college students who confront numerous challenges and transitions during their higher education journey. This research aims to investigate the influence of positive childhood experiences on the adaptability of college students and the underlying mechanisms.

**Methods:**

A cross-sectional survey was conducted with 5,787 college students from Chinese universities. Participants completed an online questionnaire assessing positive childhood experiences, self-esteem, the number of siblings, and China college students’ adjustment.

**Results:**

The results revealed that positive childhood experiences positively predicted the adjustment of China college students, with self-esteem playing a mediating role in this positive effect. Moreover, siblings played an antagonistic moderating role in the positive effect of positive childhood experiences on China college students’ adjustment.

**Discussion:**

It is recommended to enhance positive childhood experiences, improve self-esteem, and provide additional care and support to students from multi-sibling families to enhance China college students’ adjustment.

## 1 Introduction

Positive childhood experiences refer to positive and meaningful experiences that children have during their growth and development. These experiences may significantly impact a child’s physical, mental, cognitive, and emotional well-being, helping them become confident, independent, and responsible individuals. Effective communication and problem-solving skills are important qualities for those wishing to promote positive childhood experiences (Berkowitz and Grych, 1998). Positive childhood experiences profoundly influence a child’s development. They may help children build good social and emotional skills, develop self-confidence, self-control, and resilience, enhance their emotional intelligence and social skills, improve their creativity and problem-solving abilities, and cultivate positive attitudes and values toward life (Daines et al., 2021; Crouch et al., 2023; Woodward et al., 2023).

China college students’ adjustment is a key factor in determining a student’s success in adapting to new learning and living environments (Fang et al., 2005). It encompasses a broad spectrum of psychological, cognitive, and behavioral abilities crucial for coping with the challenges and changes during this transitional period (Fang et al., 2005). A student with strong adaptability may swiftly comprehend and adjust to the new academic and social environment, manage emotions and behaviors effectively, and maintain a positive and optimistic attitude toward adaptability. This includes skills such as self-regulation, problem-solving, coping with setbacks, adapting to changes, communication, teamwork, and innovative thinking (Stockinger et al., 2021). By developing and enhancing these skills, college students may increase their chances of success in both academic and personal aspects of their lives (Fang et al., 2005; Chi et al., 2022).

In order to address China college students’ adjustment issues, it is important to not only provide college students with help and support during their academic years, but also pay attention to their positive childhood experiences. Positive childhood experiences may have a significant impact on individual development and overall health (Baczewski et al., 2022; Hanson et al., 2022). It helps to cultivate individual adaptability and improve the quality of life and well-being of college students (Baczewski et al., 2022). Positive childhood experiences may promote an individual’s mental and emotional well-being and reduce their maladaptiveness when they face challenges in life (Baczewski et al., 2022; Hanson et al., 2022). For college students, positive experiences may enhance their social and emotional skills, self-esteem, and self-confidence, enabling them to better adapt to college life (Baczewski et al., 2022; Hanson et al., 2022). At present, first-year Chinese university students undergo psychological screening in the first semester. If this study is validated, the inclusion of inquiries pertaining to positive childhood experiences in the survey during screening might potentially enhance the precision of psychological counseling and mental health education tailored for university students. Although some progress has been made in understanding the relationship between positive childhood experiences and China college students’ adjustment, further research is necessary to gain a deeper understanding of this complex relationship.

In addition to the aforementioned studies, we cannot ignore the current reality. Currently, China has undergone a significant overhaul of its previous family planning policies, evolving from the strict allowance of only one child to a more lenient stance that permits two children, and most recently, embracing a policy that allows for up to three children (Agency, The Xinhua News, 2021). Does the change in the number of children in families bring about variations in the psychological states of university students? If there is an impact, what kind of influence does it exert?

Blake’s study (Blake, 1981) and Polit and Falbo’s meta-analysis (Polit and Falbo, 1987) both indicate that only children have social skills comparable to other children, but these studies have significant limitations (Downey et al., 2015). Further research suggests that only children do not acquire more social skills than their peers with siblings during the kindergarten to fifth-grade period (Downey et al., 2015). There is an association between friendly sibling relationships in early childhood and positive developmental outcomes and adaptive functioning in children later on (Howe et al., 2022). In other words, the quality of sibling relationships can influence a child’s later development (Howe et al., 2022). So, are there similar research conclusions in different cultural backgrounds? Furthermore, does this influence persist into a child’s adulthood and college years?

A comparative study in China and the United States reveals a negative correlation between the number of siblings and psychological well-being, although the specifics of this pattern (non-linear associations, sisters versus brothers, and closely versus widely spaced siblings) vary (Downey and Cao, 2023). Chinese scholars found that female children are more likely to be adversely affected when having younger brothers, while male children tend to benefit more from having older sisters (Liu and Jiang, 2021). This aligns with similar research conclusions showing a positive correlation between prosocial behavior and the number of siblings, especially sisters, although with slight variations in perspective (Liu et al., 2023). The study also confirms the negative correlation between the number of siblings and divorce among Chinese adults (Merry et al., 2020). However, what data patterns would emerge for the specific group of college students?

Currently, college students were born between 2000 and 2004, falling under China’s previous family planning policy during their birth years. Consequently, before 2013, a majority of them were the sole children in their families. The Chinese government introduced the “single two-child” and “comprehensive two-child” policies at the end of 2013 and 2015, respectively. Subsequently, their families started to expand, welcoming second children. As a result, these college students now assume the roles of elder siblings at home, fostering relationships with their younger brothers and sisters (Zhang, 2019; Fu et al., 2023). Does the alteration in family structure stemming from the family planning policy impact their psychological well-being, consequently affecting the adaptability of college students? We aim to address these questions through this study to some extent.

Furthermore, we hope that this study not only provides an explanation for these issues but also allows readers to understand the impact of China’s family planning policy changes on contemporary university students. This could potentially constitute a distinctive contribution from our study, effectively bridging the current knowledge gap in research on the adaptation of Chinese college students.

### 1.1 Theoretical basis

Social cognition theory posits that individuals form their knowledge and beliefs about themselves, others, and the environment through the observation and cognitive processing of the external environment. This, in turn, shapes their emotions, behaviors, and psychological processes (Bandura, 2023). Social cognitive theory posits that an individual’s social behavior and emotional responses are influenced by personal experiences and the environment (Bandura, 2023). During childhood, positive experiences may provide rich social information, aiding children in understanding social norms and interpersonal relationships, thereby enhancing their social cognitive abilities. The improvement in social cognitive abilities enables university students to more accurately assess and understand the emotions and needs of others when navigating complex social environments and relationships, facilitating better adaptation to social settings. Positive childhood experiences also contribute to the development of stronger self-awareness and self-efficacy in children. This self-awareness and self-evaluation capacity plays a crucial role in the adaptive development of university students. Positive childhood experiences may instill in children a more positive life attitude and set of values, which proves beneficial for their adaptation to societal changes and diverse cultural environments in university life.

Child development theory suggests that children undergo distinct stages of growth, and their progress at each stage is shaped by diverse factors, including biology, social culture, and individual experiences (Vygotsky, 1978). Positive experiences during childhood may shape more positive personality traits, such as self-esteem, self-confidence, optimism, and the like. These traits contribute to better adaptation of university students to the various challenges of university life. Positive childhood experiences also foster the development of children’s social skills and emotional management abilities. These skills may assist them in interacting more effectively with others and handling various emotional challenges during their university experience (Vygotsky, 1978). In accordance with this theory and prior research, positive experiences during childhood are anticipated to yield enduring positive effects on adaptive development in subsequent years.

Collectively, the theoretical foundation for predicting China college students’ adjustment based on positive childhood experiences is rooted in both social cognitive theory and child development theory. These frameworks posit that experiences during the positive childhood period may positively influence individuals’ social cognitive abilities and physical and mental development, thereby enhancing their adaptive development in subsequent years. Expanding upon previous research and the theoretical underpinnings, this study formulates Hypothesis 1: Positive childhood experiences predict China college students’ adjustment.

### 1.2 The mediating mechanism of self-esteem

Self-esteem is a critical aspect of an individual’s mental health and their ability to adapt to society. In research, self-esteem is often employed as a mediating variable (Peng et al., 2019; Fu et al., 2021; Liu et al., 2021). Recent studies have indicated that the relationship between adverse childhood experiences (ACE) and China college students’ adjustment is mediated by self-esteem (Rosecrance, 2022). Given the contrasting characteristics of positive childhood experiences and ACE (Baglivio and Wolff, 2021), examining the mediating effect of self-esteem between positive childhood experiences and China college students’ adjustment may provide a deeper understanding of the relationship among self-esteem, positive childhood experiences, and China college students’ adjustment. This, in turn, offers a scientific basis for promoting college students’ mental health and development. Specifically, may positive childhood experiences enhance the development of self-esteem, and may self-esteem promote the development of China college students’ adjustment? Does self-esteem mediate the relationship between positive childhood experiences and China college students’ adjustment? Therefore, this research proposes Hypothesis 2, which requires further verification: self-esteem mediates the relationship between positive childhood experiences and China college students’ adjustment.

### 1.3 The moderation mechanism of sibling

In recent decades, China has witnessed a decline in fertility rates (Yang et al., 2022). Despite the government’s efforts to encourage childbirth through policy relaxation, the total population still recorded negative growth in 2022 (China, National Bureau of Statistics, 2023). In China, college students have navigated through various policies throughout their upbringing, including the one-child policy, the comprehensive two-child policy, and the comprehensive three-child policy. As a result of these policy shifts, their roles and status within their families are subject to change. Previous studies have affirmed the moderating effect of the number of siblings on adolescent-related issues (Hoyer et al., 2021; Yang et al., 2022). Drawing on social cognitive theory and child development theory, we hypothesize that the number of siblings may play a moderating role in the relationship between positive childhood experiences and China college students’ adjustment. More precisely, the quantity of siblings may have an impact on children’s psychological attributes, encompassing social skills and self-adjustment abilities, subsequently influencing the adaptability of Chinese college students. Within families characterized by a greater number of siblings, children might be predisposed to derive advantages from positive childhood experiences, facilitating a smoother transition to college life. Conversely, in families with fewer siblings, children may lean more heavily on self-exploration and the cultivation of adaptive skills, potentially shaping the extent to which they draw benefits from positive childhood experiences. Therefore, the number of siblings may serve as a moderating factor in the correlation between positive childhood experiences and the adaptability of Chinese college students. Further empirical research is needed to investigate the mechanism by which the number of siblings moderates the influence of positive childhood experiences on China college students’ adjustment. Based on this, we propose Hypothesis 3, which awaits further verification: The number of siblings plays a moderating role between positive childhood experiences and China college students’ adjustment.

## 2 Methodology

### 2.1 Description of the tested samples

This research was ethically approved by the Academic Committee of Shandong First Medical University and reviewed by the Ethics Committee of Shandong First Medical University (approval number: R202209130117). All methods were performed in accordance with the relevant guidelines and regulations. The research utilized a sampling method to conduct a questionnaire survey in dozens of universities, with a total of 6,000 questionnaires distributed. Out of these, 5,787 valid questionnaires were recovered, resulting in an effective recovery rate of 96.45%. The research participants were college students, consisting of 2,775 males, accounting for 47.95%, and 3,012 females, accounting for 52.05%. The average age of the participants was 19.45 ± 0.32 years old. Of the participants, 5,732 were undergraduates, accounting for 99.05%, 41 were postgraduates, accounting for 0.71%, and 14 were doctoral students, accounting for 0.24%. Based on their majors, 30 were from philosophy, accounting for 0.52%, 211 were from economics, accounting for 3.65%, 24 were from law, accounting for 0.41%, 323 were from education, accounting for 5.58%, 157 were from literature, accounting for 2.71%, 4 were in history, accounting for 0.07%, 300 were from science, accounting for 5.18%, 1 was in agriculture, accounting for 0.02%, 774 were in engineering, accounting for 13.37%, 2,777 were in medicine, accounting for 47.99%, 934 were in management, accounting for 16.14%, and 252 were art students, accounting for 4.35%. The research included 5,627 Han, accounting for 97.24%, and 160 ethnic minorities, accounting for 2.76%.

### 2.2 Measures

#### 2.2.1 Positive childhood experiences Scale

For this research, the Narayan et al. (2018) scale for positive childhood experiences was utilized, which consists of 10 items. Examples of items include “Did you have at least one caregiver with whom you felt safe?” and “Did you have beliefs that gave you comfort?” Response options ranged from 1 (strongly disagree) to 5 (strongly agree). The scale’s reliability was evaluated using Cronbach’s α coefficient, which was found to be 0.88, indicating high internal consistency. The scale’s validity was also assessed using the KMO value, which was found to be 0.92, indicating that the scale was valid for further analysis. Furthermore, the Bartlett test was conducted to confirm the reliability and validity of the scale.

#### 2.2.2 Self-esteem Scale

In this research, the Rosenberg self-esteem Scale (Rosenberg, 1965) was utilized, which includes 10 items such as “I have a positive attitude toward myself,” “I hope to win more respect for myself,” and “In general, I am satisfied with myself,” with reverse items such as “I sometimes feel that I am useless.” The same five-point Likert scale was used, ranging from 1 (strongly disagree) to 5 (strongly agree), with higher scores indicating higher levels of self-esteem. Data processing was performed on the reverse items of the scale before data analysis. The Cronbach’s α coefficient for the scale was 0.85, indicating good internal consistency, and the KMO value for the validity was 0.86. The reliability and validity of the scale meet the requirements for further analysis, as confirmed by the Bartlett test.

#### 2.2.3 China college students’ adjustment Scale

This research utilized the Chinese College Student Adaptation Scale (Fang et al., 2005), developed by Fang et al. and the research team of the College Student Mental Health Evaluation System of the Ministry of Education of China. The scale includes six dimensions and 51 items, namely interpersonal relationship adaptation (e.g., “Many people ask me to play with them”), learning adaptation (e.g., “I usually read books related to my major”), campus life adaptation (e.g., “I have a rich spare time life, and I do not need to do anything to change”), emotional adaptation (e.g., “There are always things I am interested in in my daily life”), self-adaptation (e.g., “I think my advantages are more than disadvantages”), and satisfaction (e.g., “If I were asked to choose again, I would still live as it is now”). The scale uses a five-point Likert scoring method ranging from 1 (strongly disagree) to 5 (strongly agree), including reverse scoring items (e.g., “When I encounter discouraging things, I often feel helpless”). The scale has a high level of reliability, with a measured Cronbach’s α coefficient of 0.95, and high validity, with a KMO value of 0.98. The scale meets the requirements for further analysis based on the results of the Bartlett test.

The number of siblings is a categorical variable with four levels, coded as 1 for only children (indicating that they have no siblings), 2 for eldest siblings with younger brothers/sisters (indicating they have at least one younger sibling), 3 for younger siblings with an older brother/sister (indicating they have at least one older sibling), and 4 for those with both an older and younger brother/sister (indicating they have at least two siblings).

### 2.3 Data collection procedure

To facilitate the distribution of scales, the research team employed the electronic network questionnaire form provided by Questionnaire Star to generate a link. The researchers shared the QR code of the scale with university teachers and students, who, in turn, forwarded it to other students. During the data collection process for this research, some colleges and universities were in lockdown due to the epidemic, making face-to-face questionnaire completion inconvenient. Therefore, the electronic questionnaire format was adopted.

Following the research paradigm, the scales were presented to participants in the order of informed consent (explaining the significance and value of the research), personal basic information, Positive Childhood Experiences scale, self-esteem scale, and Chinese college students’ adjustment scale. At the beginning of the questionnaire, an informed consent form was included, requiring participants to read and agree before proceeding with the survey. Failure to do so resulted in the termination of the survey. Two pilot tests were conducted before the formal scale test. Participants were not remunerated for filling out the scales; all participation was voluntary, taking approximately 10 min to complete. The researchers expressed their gratitude to the participating students at the end of the research.

### 2.4 Data analysis process

In this research, data analysis and hypothesis testing were conducted using the online analysis software SPSSAU (SPSSAU, 2023). Initially, descriptive statistics, confirmatory factor analysis, normality tests, and Pearson correlation analysis were performed on the samples. Subsequently, regression analysis was conducted on the control variables (age, gender, grade, and major), predictor variables, mediator variables, moderator variables, and outcome variables to verify the main effects. The mediating effect of self-esteem was analyzed using Hays’ method (Hayes, 2013), and verification was performed through the bootstrap method. Furthermore, the analysis of the moderator effect of the categorical variable ‘Sibling’ was carried out (Hayes and Matthes, 2009; Fang and Wen, 2022), and a simple slope diagram was created.

### 2.5 Common method bias

In this research, data were collected online from college students who used their mobile phones or computers to answer questions. Recognizing the potential impact of the data source, measurement environment, and project context on common method bias, a test was crucial to ensure the accuracy of the statistical results. Harman’s single-factor method was employed for this purpose. Specifically, all items underwent exploratory factor analysis, and the results revealed that the first factor without rotation explained only 29.9% of the total variation, which is below the critical value of 40%. Based on these findings, it might be concluded that there is no significant common method bias in this research (Yang et al., 2022).

## 3 Results

### 3.1 Descriptive statistics

The overall model of this research was confirmed by a factor analysis with satisfactory results: *χ*^2^/df = 3.97, GFI = 0.93, RMSEA = 0.07, RMR = 0.03, CFI = 0.95, NFI = 0.95, and NNFI = 0.95. These results met the criteria for further analysis. To test for normality, the Kolmogorov–Smirnov test was performed on the data (Lilliefors, 1967; Drezner et al., 2010). The results showed that the absolute value of kurtosis was less than 10 and the absolute value of skewness was less than 3. Although the data was not entirely normally distributed, it was deemed acceptable for normal distribution. Pearson correlation analysis was conducted on the variables, and Table 1 shows that positive childhood experiences is significantly and positively correlated with the six dimensions of self-esteem and China college students’ adjustment. Additionally, the six dimensions of self-esteem and China college students’ adjustment are significantly and positively correlated with each other.

**Table 1:** Correlation analysis of variables.

	Mean value	Standard deviation	1	2	3	4	5	6	7	8	9	10	11	12	13	14
Sex (1)	1.520	0.500	1													
Age (2)	1.280	0.482	0.011	1												
Grade (3)	1.665	0.864	0.081***	0.632***	1											
Magor (4)	9.118	2.411	−0.085***	−0.157***	−0.243***	1										
Ethnic (5)	1.028	0.164	0.037**	0.064***	0.043***	−0.039**	1									
Sibling (6)	2.042	0.899	0.086***	0.013	0.014	−0.074***	−0.003	1								
Positive childhood experiences (7)	4.016	0.727	−0.065***	−0.048***	−0.058***	0.080***	−0.065***	−0.076***	1							
SE (8)	2.052	0.614	0.071***	−0.016	0.001	0.014	−0.046***	−0.032*	0.532***	1						
Campus life adaptation (9)	1.489	0.610	−0.010	−0.048***	−0.061***	0.035**	−0.037**	−0.078***	0.487***	0.555***	1					
Learning adaptation (10)	1.901	0.627	−0.008	−0.015	−0.024	0.048***	−0.050***	−0.059***	0.480***	0.548***	0.729***	1				
Self-adaptation (11)	1.546	0.500	−0.010	0.002	0.018	0.011	−0.054***	−0.043**	0.483***	0.707***	0.684***	0.692***	1			
Satisfaction (12)	2.384	0.702	−0.067***	−0.063***	−0.079***	0.075***	−0.052***	−0.071***	0.481***	0.513***	0.713***	0.688***	0.650***	1		
Interpersonal relationship adaptation (13)	1.893	0.629	0.039**	0.002	0.013	0.018	−0.037**	−0.065***	0.518***	0.592***	0.696***	0.664***	0.719***	0.602***	1	
Emotional adaptation (14)	2.374	0.616	−0.038**	−0.031*	−0.042**	0.044***	−0.045***	−0.049***	0.557***	0.628***	0.765***	0.737***	0.777***	0.764***	0.723***	1

*p < 0.05; **p < 0.01; ***p < 0.001.

### 3.2 Mediating effect analysis of self-esteem

The mediating effect of self-esteem is analyzed through three models, as presented in Table 2. The first model, China college students’ adjustment = 0.232 + 0.423positive childhood experiences, shows that positive childhood experiences has a significant positive effect on China college students’ adjustment (*t* = 53.694, *p* = 0.000 < 0.05), which supports Hypothesis 1. The second model, self-esteem = 0.248 + 0.449 positive childhood experiences, shows that positive childhood experiences also has a significant positive effect on self-esteem (*t* = 47.828, *p* = 0.000 < 0.05). The third model, China college students’ adjustment = 0.122 + 0.224 positive childhood experiences +0.444 self-esteem, indicates that both positive childhood experiences and self-esteem have a significant positive effect on China college students’ adjustment, and the effect of self-esteem on China college students’ adjustment is also significant (*t* = 47.466, *p* = 0.000 < 0.05). This supports the mediating effect of self-esteem on the relationship between positive childhood experiences and China college students’ adjustment, validating Hypothesis 2.

**Table 2:** Mediating effect test model.

	China college students’ adjustment	Self-esteem	China college students’ adjustment
Constant	0.232***(7.206)	0.248***(6.462)	0.122***(4.444)
Positive childhood experiences	0.423***(53.694)	0.449***(47.828)	0.224***(28.308)
Self-esteem			0.444***(47.466)
Sample size	5,787	5,787	5,787
*R*^2^	0.333	0.283	0.520
Adjusted *R* 2	0.332	0.283	0.520
F	*F* (1,5,785) = 2883.044, *p* = 0.000	*F* (1,5,785) = 2287.526, *p* = 0.000	*F* (2,5,784) = 3129.201, *p* = 0.000

*p < 0.05; **p < 0.01; ***p < 0.001.The value of *t* is shown in parentheses.

The indicators used in the mediating analysis of this research are explained in Table 3. The value *c* = 0.423*** represents the regression coefficient of X to Y, which is the total effect when there is no mediating variable M in the model. The value *a* = 0.449*** indicates the regression coefficient of X to M, while *b* = 0.444*** indicates the regression coefficient of M to Y. The product of a and b, which is ab = 0.200, represents the indirect effect or mediating effect. The value *c*’ = 0.224** indicates the regression coefficient of X to Y when a mediating variable M is present in the model, representing the direct effect.

**Table 3:** Effects of mediating.

Term	c	a	b	a*b	a*b (95% BootCI)	c’	Conclusion
Positive childhood experiences= > Self-esteem= > China college students’ adjustment	0.423***	0.449***	0.444***	0.200	0.257 ~ 0.288	0.224***	Partial mediating

*p < 0.05; **p < 0.01; ***p < 0.001.

The analysis results demonstrate that both the coefficients of a and b are significant, as well as c’. The product of a and b has the same sign as c’, and the 95% BootCI confidence interval [0.257 ~ 0.288], calculated through Bootstrap sampling, does not include 0. Therefore, self-esteem plays a partial mediating role, with the mediating effect accounting for 47.16% of a*b/c. The 95% confidence intervals for each effect value of mediating do not include 0. Therefore, Hypothesis 2 is confirmed.

### 3.3 Sibling’s moderating effect test

In this research, a moderation analysis was conducted to examine the effect of the moderating variable (Sibling) on the relationship between the independent variable (positive childhood experiences) and the dependent variable (China college students’ adjustment). The analysis involved three models (Table 4). Model 1 examined the relationship between positive childhood experiences and China college students’ adjustment without considering the moderating effect of Sibling. In Model 2, Sibling was added as an adjustment variable. Finally, in Model 3, an interaction term between positive childhood experiences and Sibling was included to test for moderation effects.

**Table 4:** Results of moderation analysis.

	Model 1	Model 2	Model 3
Constant	1.931*** (337.082)	1.947*** (191.280)	1.945*** (191.139)
Positive childhood experiences	0.423*** (53.694)	0.422*** (53.374)	0.464*** (33.207)
Sibling-1.0 [Reference Item]	–	–	–
Sibling-2.0		−0.007 (−0.534)	−0.005 (−0.400)
Sibling-3.0		−0.054*** (−3.444)	−0.055*** (−3.467)
Sibling-4.0		−0.011 (−0.469)	−0.021 (−0.846)
Positive childhood experiences*Sibling-2.0			−0.042* (−2.191)
Positive childhood experiences*Sibling-3.0			−0.078*** (−3.696)
Positive childhood experiences*Sibling-4.0			−0.112*** (−3.407)
Sample size	5,787	5,787	5,787
*R*^2^	0.333	0.334	0.336
Adjusted *R* ^2^	0.332	0.334	0.336
*F*	*F* (1,5,785) = 2883.044, *p* = 0.000	*F* (4,5,782) = 725.454, *p* = 0.000	*F* (7,5,779) = 418.575, *p* = 0.000
△*R*^2^	0.333	0.002	0.002
△*F*	*F* (1,5,785) = 2883.044, *p* = 0.000	*F* (3,5,782) = 4.508, *p* = 0.004	*F* (3,5,779) = 6.596, *p* = 0.000

Dependent variable China college students’ adjustment.

*p < 0.05; **p < 0.01; ***p < 0.001, *t*-value in parentheses.

**Figure 1:**
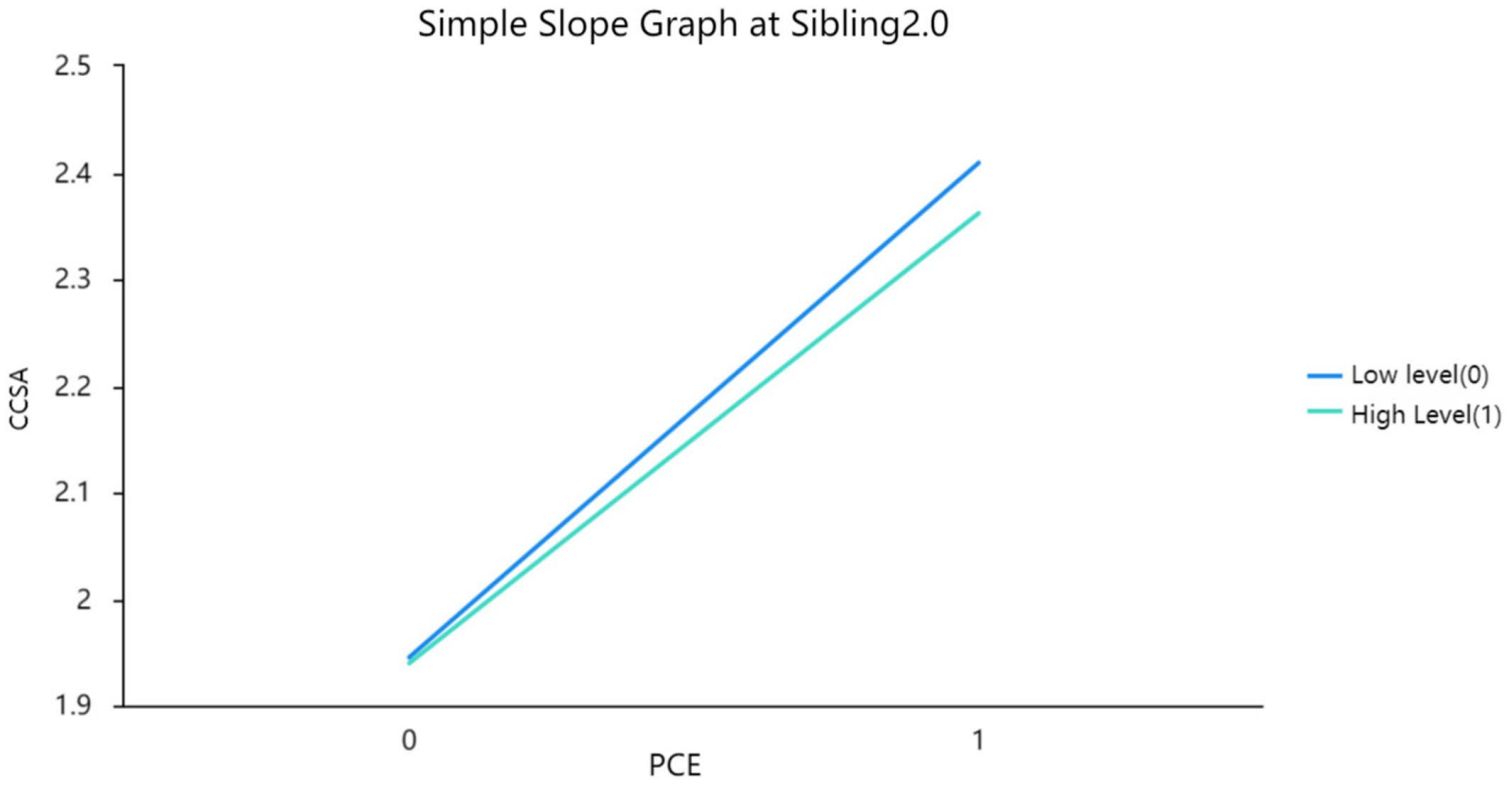
Simple slope graph at sibling 2.0.

**Figure 2:**
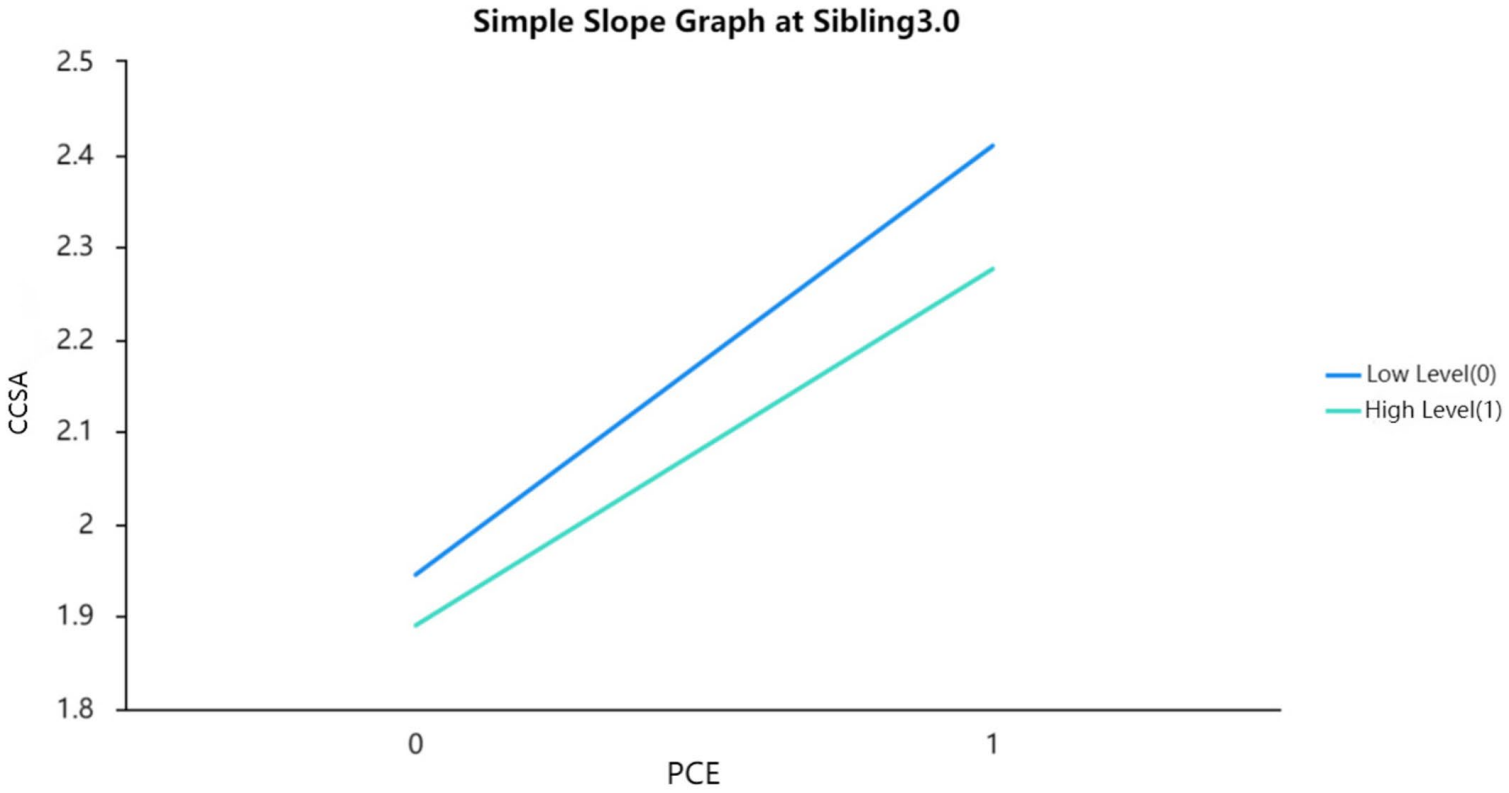
Simple slope graph at sibling 3.0.

**Figure 3:**
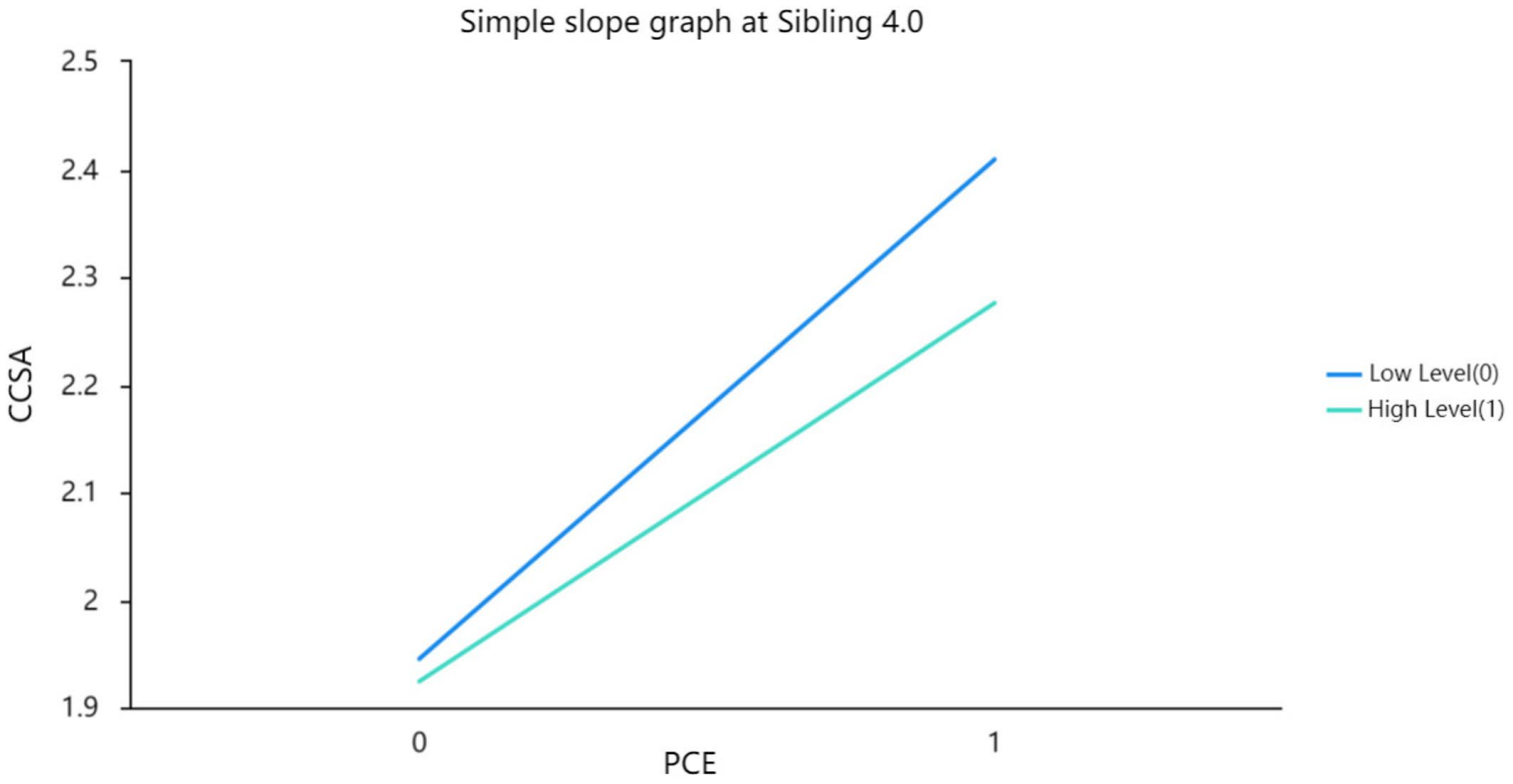
Simple slope graph at sibling 4.0.

The researchers found that the interaction terms between positive childhood experiences and Sibling in Model 3 were significant, suggesting that the moderating effect of Sibling on the relationship between positive childhood experiences and China college students’ adjustment was statistically significant. They then used a simple slope graph to visually display the magnitude of the effect of positive childhood experiences on China college students’ adjustment at different levels of sibling (see Figures 1–3). Based on their analysis, they confirmed Hypothesis 4.

It is worth noting that the researchers used dummy variables to represent sibling in their analysis, and the dependent variable (China college students’ adjustment) was not transformed in any way. Overall, the research appears to have employed a rigorous approach to examining the moderating effect of Sibling on the relationship between positive childhood experiences and China college students’ adjustment.

## 4 Conclusion and discussion

The data analysis in this research revealed that positive childhood experiences is a significant predictor of China college students’ adjustment, and that self-esteem plays a mediating role in the relationship between positive childhood experiences and China college students’ adjustment. Additionally, the research found that Sibling moderates the predictive effect of positive childhood experiences on China college students’ adjustment. These findings confirm all three hypotheses of the research, which will be discussed in further detail below.

### 4.1 About positive childhood experiences predicting China college students’ adjustment

The first key finding of this research supports social cognitive theory and child development theory, consistent with prior studies by other scholars (Vygotsky, 1978; Huang and Fuming, 2004; Bandura, 2023), indicating that positive childhood experiences positively impact an individual’s social cognitive ability and physical and mental development, leading to enhanced levels of adaptive development. It is crucial to note, however, that different types of positive childhood experiences may exert varying effects on different dimensions of China college students’ adjustment. For example, sports experience may be more beneficial for emotional, while social practice may be more conducive to learning. Therefore, further empirical research is needed to explore the relationship between different types of positive childhood experiences and the various dimensions of China college students’ adjustment.

The second key finding highlights the need for further exploration of the causal relationship between positive childhood experiences and China college students’ adjustment. Positive childhood experiences may contribute to Chinese university students’ better adaptation to the college environment. Firstly, positive childhood experiences may cultivate students’ emotional adaptation skills. During childhood, students go through interactions with family, friends, and teachers, learning how to express their emotions and needs while understanding the emotions of others. This emotional adaptation skill is equally crucial in the college environment, where students need to establish good relationships with roommates, peers, and professors, and adapt to academic and social pressures. Secondly, positive childhood experiences may enhance students’ social skills. During childhood, students learn how to connect, communicate, and cooperate with others. These skills are also vital in the college environment, where students need to build good relationships with roommates, peers, and professors, and adapt to academic and social pressures. Finally, positive childhood experiences may foster students’ psychological resilience. When facing challenges and setbacks, students with positive childhood experiences may find it easier to cope and adapt because they have learned how to handle stress and overcome difficulties. This psychological resilience is equally crucial in the college phase, where students need to navigate various academic and social challenges.

While this research identified a predictive relationship between positive childhood experiences and China college students’ adjustment, establishing causality requires further investigation. Randomized controlled trials and sophisticated research designs will be necessary for this purpose. Moreover, it’s crucial to consider other influencing factors such as social support and family environment, and their interactions with positive childhood experiences need exploration to elucidate the predictive mechanism of positive childhood experiences on China college students’ adjustment. Future research should also delve into the long-term impact of positive childhood experiences on China college students’ adjustment, considering potential variations across different life stages.

Lastly, it is essential to explore the generalizability of the findings from this research across various cultural and social contexts, encompassing factors such as gender, age, family background, and socioeconomic status. Additionally, repeated studies conducted by different scholars are necessary to affirm the reliability of the predictive relationship between positive childhood experiences and China college students’ adjustment. In summary, while this research affirms the predictive connection between positive childhood experiences and China college students’ adjustment, further research is imperative to offer more comprehensive guidance for education and mental health interventions.

### 4.2 About the mediating effect of self-esteem

Firstly, this research suggests that self-esteem plays a mediating role in the relationship between positive childhood experiences and China college students’ adjustment. In other words, positive childhood experiences may shape an individual’s self-esteem, thereby influencing their adaptive capacity. This impact may be realized through various pathways, such as fostering self-confidence, problem-solving abilities, and cultivating a positive attitude toward new environments. It indicates that positive childhood experiences may enhance China college students’ adjustment by increasing self-esteem. Self-esteem is influenced by various factors such as individual character, personality, growth environment, and family education (Fang et al., 2016; Zhang et al., 2020; Nan and Wang, 2022). Low self-esteem may lead to negative emotions such as anxiety and depression, while high self-esteem may make individuals more confident, optimistic, and positive (Fang et al., 2016; Sang et al., 2019). In childhood, factors such as parental rearing style, family environment, and interaction with peers have a profound impact on the formation of self-esteem (Fang et al., 2016; Yang et al., 2021). It is important to consider the individual’s growth environment, development track, and change laws of self-esteem when discussing the mediating role of self-esteem (Bao et al., 2016; Fang et al., 2016).

Secondly, the mediating effect of self-esteem is not unidirectional, and other factors may affect self-esteem and thus the mediating effect of self-esteem between positive childhood experiences and China college students’ adjustment (Bao et al., 2016). Other mediator variables or moderator variables, such as discrimination intuition (Bao et al., 2016) or psychological resilience (Chai et al., 2018), may exist. Future research needs to explore this issue more deeply to improve our understanding of the mechanism of self-esteem in mental health and adaptive development. This research provides a scientific basis for conducting relevant interventions and improving China college students’ adjustment by helping us better understand the mechanism of self-esteem.

### 4.3 Regarding the moderating effect of sibling

This research provides important insights into the complex relationships among positive childhood experiences, self-esteem, sibling relationships, and China college students’ adjustment. The findings suggest that positive childhood experiences may have a significant impact on an individual’s adaptive development, particularly through its effects on self-esteem. The research underscores the significance of factoring in the number of siblings in a family when evaluating an individual’s growth and development. This is particularly crucial, as having multiple siblings may potentially decelerate or diminish the impact of positive childhood experiences on the adjustment of Chinese college students.

Drawing upon social cognitive theory, it is reasonable to posit that the presence of siblings might enhance opportunities for social learning, facilitated through the observation of sibling interactions. Consequently, the potential existence of siblings may actively contribute to the development of social skills and other forms of knowledge, ultimately enhancing the adaptability of college students. However, the empirical results obtained are contrary to this expectation.

Scholarly studies conducted previously using ECLS-K data documented social skills deficits (interpersonal skills, self-control, and externalizing problem behaviors, etc.) in American only children upon entering kindergarten (Downey and Condron, 2004). Downey et al.’s (2015) research advanced this line of inquiry by demonstrating that these deficits did not significantly change even after 5 years of school education, highlighting the persistence of these issues (Downey et al., 2015). Our similar study further establishes that the impact of these deficits continues into college. Specifically, Downey et al.’s (2015) findings suggest that the early gaps observed in only children during kindergarten may be challenging to bridge (Downey et al., 2015), even after 5 years of interaction with peers in school, and this effect persists into college. This further supports Yucel’s (2011) assertion that, in the process of child development, it is crucial to focus not only on the number of siblings but also on the quality of sibling relationships (Yucel, 2011). Perhaps, the emergence of such research conclusions is due to the quality of sibling relationships? If so, our study findings will also to some extent corroborate the viewpoint presented earlier by Howe et al. (2022), emphasizing that the quality of sibling relationships impacts a child’s future development, including adaptability in college.

In order to provide a clearer explanation, we turned to Chinese scholars’ research for insights. Primarily, with the addition of a second child to the family, the original family structure and roles undergo transformations, exerting a certain impact on the emotional and behavioral expressions of the older child. Studies indicate that the arrival of a second child affects the older sibling, leading to the experience of negative emotions. This phenomenon may stem from the slight disruption of their central position, resulting in a reduction in attention and expectations from the family (Fang, 2018; Zhang, 2019).

Secondly, the introduction of a second child may indeed stimulate increased sibling interaction among brothers and sisters, but it may also engender certain adverse effects on the behavior of the older child. Research has demonstrated that the addition of new family members disrupts the psychological framework of the older child within the original family. While adapting to the evolving family structure, the older child encounters challenges such as feelings of depression, competition for attention with younger siblings, and a decline in self-care abilities (Li, 2017; Zhang, 2019).

Finally, the decision to expand the family by having a second child is not only a demographic consideration for population growth but also a strategic move in optimizing family structure. It plays a vital role in fostering the socialization of the older child. Parents, in this context, should prioritize both the optimization of family structure and the holistic development of the older child’s physical and mental well-being. In the realm of family life and parenting, the older child, when psychologically prepared for the arrival of a sibling, may actively contribute to achieving this dual objective. Parents, recognizing the transformative role and psychological impact of the second child, should earnestly attend to the individual needs of the older child. Guiding them thoughtfully, parents may help them navigate and embrace the changes accompanying the arrival of the second child, fostering acceptance and adaptation (Zhang, 2019).

Upon deeper analysis, is this a profound societal issue stemming from the changes in the family planning policy? The implications of these findings extend beyond the college context and have important implications for family, educational, and social policy. Future research is needed to further explore these relationships and develop effective interventions and policies to promote positive outcomes for individuals and families.

Furthermore, the findings of this research have implications for parenting and education in general. It is crucial to consider the impact of the number of siblings in a family on children’s cognitive, emotional, and social adaptation. Parents and educators should pay attention to the upbringing of children in families with multiple siblings, understanding the impact of childhood experiences on self-esteem and adaptive abilities. They should strive to provide positive childhood experiences as much as possible to aid children in developing self-esteem and confidence. Parents should strive to balance the allocation of resources and ensure that each child receives enough support and attention to promote their individual growth and development. Similarly, schools and teachers should be aware of the potential challenges and competition among students with multiple siblings and provide them with appropriate support and attention to help them adjust to the school and social environment. By taking steps to encourage positive interactions and cooperation among siblings, parents and educators may promote a healthy and supportive family and educational environment for children. Furthermore, social policies should be adjusted based on these findings to support university students from families with multiple siblings. For instance, providing additional resources such as psychological counseling or academic support may assist these students in better adapting to university life.

The results of this research shed light on the interplay between positive childhood experiences, self-esteem, sibling relationships, and China college students’ adjustment, highlighting the importance of individual experiences and characteristics in the assessment and promotion of mental health and adaptive development. These findings have significant implications for family, educational, and social policies, emphasizing the need for tailored interventions and policies to promote positive outcomes for individuals and families. Further research is needed to explore the mechanisms underlying the effects of positive childhood experiences and sibling relationships on China college students’ adjustment, which may inform the development of effective interventions and policies.

### 4.4 Research limitations and practical application prospects

The limitations of this research should also be taken into consideration. The use of cross-sectional data may limit the ability to establish causal relationships between variables. Additionally, self-report questionnaires may introduce response biases and social desirability effects. Additionally, the high proportion of medical students and the lockdown measures resulting from the COVID-19 pandemic might potentially impact the psychological state of university students, leading to potential discrepancies in self-report data. Additionally, as described in the preceding sections, the uniqueness of our study sample, composed of college students influenced by social policies, necessitates consideration of the distinct cultural, demographic, or societal factors inherent in the sample. This may to some extent confer uniqueness to our study. In discussing the study, we compared the research results with previous studies and took into account crucial social contextual factors. It would be beneficial for future research to conduct replication studies to validate the generalizability of the findings.

Despite these limitations, the research underscores the crucial role of promoting self-esteem and supporting college students’ adaptation. From an educational perspective, creating positive experiences and nurturing environments for children is essential for cultivating self-esteem and ensuring their success in adapting to new environments. Furthermore, the research lays the groundwork for future studies in psychology, pedagogy, sociology, and related fields to delve into the intricate relationship between self-esteem, positive childhood experiences, and college student adaptation. Subsequent experimental research may delve deeper into the underlying mechanisms that influence the impact of positive childhood experiences on college student adaptation, thereby enhancing the practicality and reliability of the findings.

## Data availability statement

The original contributions presented in the study are included in the article/supplementary material, further inquiries can be directed to the corresponding author.

## Ethics statement

The studies involving humans were approved by the Ethics Committee of Shandong First Medical University. The studies were conducted in accordance with the local legislation and institutional requirements. The participants provided their written informed consent to participate in this study.

## Author contributions

JL: Writing – original draft, Writing – review & editing, Conceptualization, Data curation, Formal analysis, Investigation. XZ: Methodology. CZ: Data curation. WW: Data curation, Investigation, Writing – review & editing. SC: Writing – review & editing.
